# Two cases of colonic tumors observed by linked color imaging and texture and color enhancement imaging with the tablet‐image comparison method

**DOI:** 10.1002/deo2.47

**Published:** 2021-08-24

**Authors:** Yuri Tomita, Naohisa Yoshida, Ken Inoue, Hikaru Hashimoto, Satoshi Sugino, Ryohei Hirose, Osamu Dohi, Yoshito Itoh

**Affiliations:** ^1^ Department of Molecular Gastroenterology and Hepatology Graduate School of Medical Science Kyoto Prefectural University of Medicine Kyoto Japan

**Keywords:** colonoscopy, high‐grade dysplasia, linked color imaging, texture and color enhancement imaging

## Abstract

An endoscope system using 5‐color light‐emitting diodes (LEDs) (EVIS X1: CV‐1500, Olympus Co., Tokyo, Japan) was released worldwide in July 2020. In addition to the improvement of narrow band imaging (NBI), this system enables texture and color enhancement imaging (TXI). TXI makes the lesion reddish and supports better visibility of colorectal lesions in comparison to white light imaging for improving lesion detection. On the other hand, another 4‐color LED endoscope system (ELUXEO: BL‐7000; Fujifilm, Tokyo, Japan) has been on the market in the West since 2017. This system enables blue light imaging (BLI) and linked color imaging (LCI). Generally, the accurate comparison between two images obtained by two different endoscope systems is difficult. To resolve this problem, we developed a method named the tablet‐image comparison (TIC) method. TIC is a simple, easy, and paperless method to get images under similar conditions of two endoscope systems for an accurate comparison. We herein report two colorectal lesions in which accurate comparisons of images between TXI and LCI and between improved NBI and BLI obtained in the EVIS X1 and ELUXEO systems were performed using the TIC method. One was IIa 30 mm (high‐grade dysplasia) and the other was IIa 25 mm (low‐grade adenoma). A detailed comparison between TXI and LCI could be performed by TIC. In these two cases, with a distant view, TXI showed greater redness than LCI. LCI showed slightly higher brightness than TXI. In magnified TXI and LCI, the irregularities observed were similar to NBI and BLI, respectively.

## INTRODUCTION

A novel endoscope system that uses light‐emitting diodes (LEDs) of five colors as a light source (EVIS X1: CV‐1500; Olympus Co., Tokyo, Japan) and adopts both the simultaneous method and the frame sequential method was released worldwide in July 2020. A dedicated colonoscope with a dual focus function (CF‐EZ1500DL/I) under the simultaneous method was also released (Table 1). The five colors of the LEDs are blue, blue‐violet, green, amber, and red, and the endoscope is equipped with several new technologies, such as an extended depth of field (EDOF), red dichromatic imaging (RDI), and texture and color enhancement imaging (TXI), as well as narrow band imaging (NBI) and white light imaging (WLI).^1^, ^2^, ^3^, ^4^ In November 2021, a manually magnified endoscope for EVIS X1 that incorporates these technologies (CF‐XZ1200L/I), which can magnify lesions 135 times and use the frame sequential method was released. The images captured by NBI are brighter and clearer in comparison to previous systems. TXI is designed to enhance three image factors (texture, brightness, and color) by applying the retinex theory.^1^ The basis of the TXI algorithm, the image obtained from WLI is divided into a base image and a texture image. Then, texture and brightness are adjusted to these two images and they are combined. After that, color enhancement is performed to the image.

**TABLE 1:** Comparison of EVIS X1 and ELUXEO

	EVIS X1	ELUXEO
System	CV‐1500	BL‐7000 (light) VP‐7000 (processor)
Dedicated colonoscopy	CF‐EZ1500DL/I (Simultaneous method) CF‐XZ1200L/I (Frame sequential method)	EC‐760ZP‐V/M (Simultaneous method)
Color of LED	5 colors (violet, blue, green, amber, red)	4 colors (blue‐violet, blue, green, red)
Our setting		
WLI	A5	H/+4/+4
NBI/BLI	A8	B8/C2
TXI/LCI	Moderate	B8/C3

Abbreviations: BLI, blue light imaging; LCI, linked color imaging; NBI, narrow‐band imaging; TXI, texture and color enhancement imaging; WLI, white light imaging.

On the other hand, a LED endoscope system using four LED lights (ELUXEO: BL‐7000; Fujifilm, Tokyo, Japan) and a dedicated colonoscope (EC‐760ZP‐V/M) was released in Europe and the United States in 2017 (Table 1). The four colors of these LED lights are violet, blue‐violet, green, and red, and the multi‐light technology that appropriately adjusts the balance and output of each light enables blue light imaging (BLI) and linked color imaging (LCI) for the detection and characterization of lesions.^5^, ^6^, ^7^ Both TXI and LCI are made to improve the visibility of lesions by making a lesion reddish and fading the surroundings. But there are image differences between these two modalities, which have not been well examined.

Generally, the comparison between two images obtained by two endoscope systems is difficult because the amount of air around a lesion, the endoscopic angle to a lesion, and the distance from a lesion vary easily. To resolve this problem, we developed our original method named the tablet‐image comparison (TIC) method. TIC is a simple method to get images under similar conditions for comparing images from two endoscope systems accurately. We herein report two cases of high‐grade colonic dysplasia in which accurate comparisons of images between TXI and LCI and between improved NBI and BLI obtained in the EVIS X1 and ELUXEO systems were performed using TIC.

## TABLET‐IMAGE COMPARISON

**FIGURE 1:**
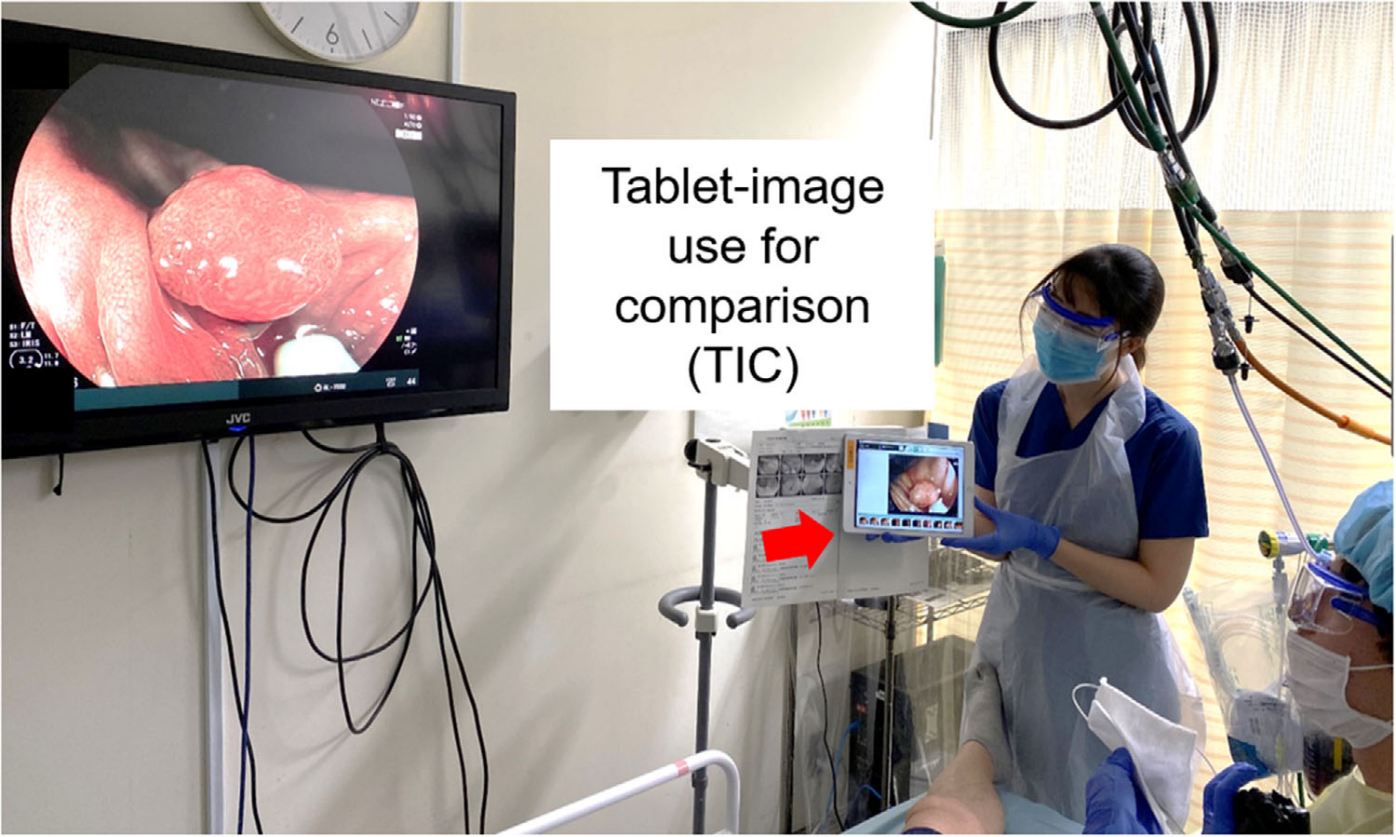
The tablet‐image comparison (TIC). Images obtained via the first modality are displayed on the tablet held next to the monitor by an assistant. According to this, the endoscopist can adjust the amount of air and the angle and distance of the lesion similarly in the second modality and produce images for the comparison of the two modalities under conditions that are largely the same

With advances in endoscope systems, the comparison of images obtained with different modalities is becoming increasingly important. We developed the TIC method, which gets images under the same conditions of two different endoscope systems in the same case for an accurate comparison. In detail, images obtained with the first system are saved in the filing system, and then, we take a picture of these images with a tablet device. Subsequently, these images are displayed on the tablet held next to the monitor of a second system by an assistant. Then, images are obtained with a second system according to the images obtained with the first modality, endoscopists can then adjust the amount of air and the angle and distance of a lesion to make the images obtained with the two modalities accurately same (Figure 1). Finally, accurate comparison about the difference of two modalities can be performed using the images obtained by TIC.

## CASE REPORTS WITH TIC

**FIGURE 2:**
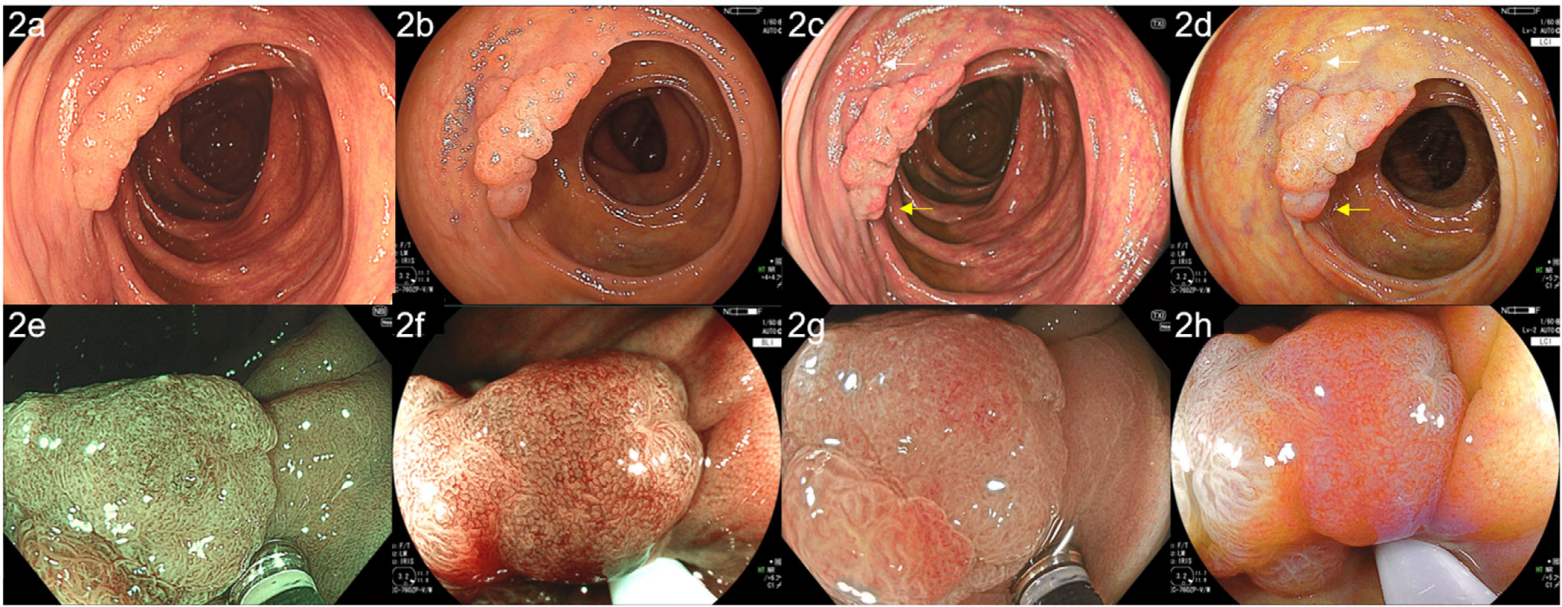
Images of high‐grade dysplasia were obtained by color enhancement imaging (TXI) and linked color imaging (LCI) using the EVIS X1 (CF‐EZ1500DI) and ELUXEO systems, compared by tablet‐image comparison (TIC). (a) A superficial elevated lesion of 30 mm with white light imaging (WLI) using CF‐EZ1500DI (high‐grade adenoma, ascending colon). (b) In WLI performed using the ELUXEO system, comparison by TIC revealed that the redness and brightness of the image were not so different from the redness and brightness on the images obtained by the EVIS X1 system. (c) On TXI, the granules of the lesion were generally reddish, and the redness was particularly emphasized at the depression (yellow arrow). A hyperplastic polyp of 2 mm could be seen (white arrow). (d) Compared to TXI, LCI suppressed the redness overall. The depressed area was more reddish in comparison to the surrounding areas (yellow arrow). Hyperplastic polyps of 2 mm could be clearly seen (white arrow). (e) Magnified narrow band imaging (NBI) clearly showed an irregular surface pattern. The vessel pattern was thin and complex. (f) An image of the same area was more brownish under magnified blue light imaging (BLI) and the surface pattern was less irregular and more enhanced in comparison to images obtained by the EVIS X1 system. (g) TXI magnified observation showed that the surface pattern was irregular and complex, similar to NBI. (h) LCI magnified observation showed that the surface pattern was irregular, similar to BLI

The first case was a woman in her 70s. A neoplastic lesion on the ascending colon was observed with EVIS X1 for the preoperative diagnosis and then with ELUXEO by TIC on the day of endoscopic submucosal dissection (ESD). A distant‐view observation with WLI using CF‐EZ1500DI (EVIS X1: structure enhancement setting: A5) revealed a superficial elevated lesion of 30 mm with granules on the surface (Figure 2a). In WLI using ELUXEO (setting: H/+4/+4) by TIC, the redness and brightness of the image were not so different from the EVIS X1 image (Figure 2b). On TXI, the granules of the lesion were generally reddish, and the redness was particularly emphasized at the depression (Figure 2c). In comparison to TXI, the suppression of redness was similar in LCI (setting: B8/C3) (Figure 2d). LCI showed slightly higher brightness. The depressed area was more reddish in comparison to surrounding areas. Magnified observation of the reddish area with NBI (structure enhancement setting: A8) clearly showed an irregular surface pattern (Figure 2e). The vessel pattern was thin and complex. An image of the same area was more brownish under magnified BLI (setting: B8/C2) and the surface pattern was less irregular and more enhanced in comparison to EVIS X1 imaging (Figure 2f). The vessel pattern was slightly thicker than with NBI and showed a clear network pattern. TXI magnified observation (TXI1) emphasized the redness (Figure 2g). The surface pattern was irregular and complex, similar to NBI. On LCI magnified observation (setting: B8/C3), the surface pattern was an irregular shape with clear edges, and the network structure of the vessel pattern was clearly confirmed to be similar to that observed by BLI (Figure 2h). We diagnosed this lesion as JNET 2B according to the findings of NBI and BLI and performed ESD.^8^ A histopathological examination revealed high‐grade dysplasia.

**FIGURE 3:**
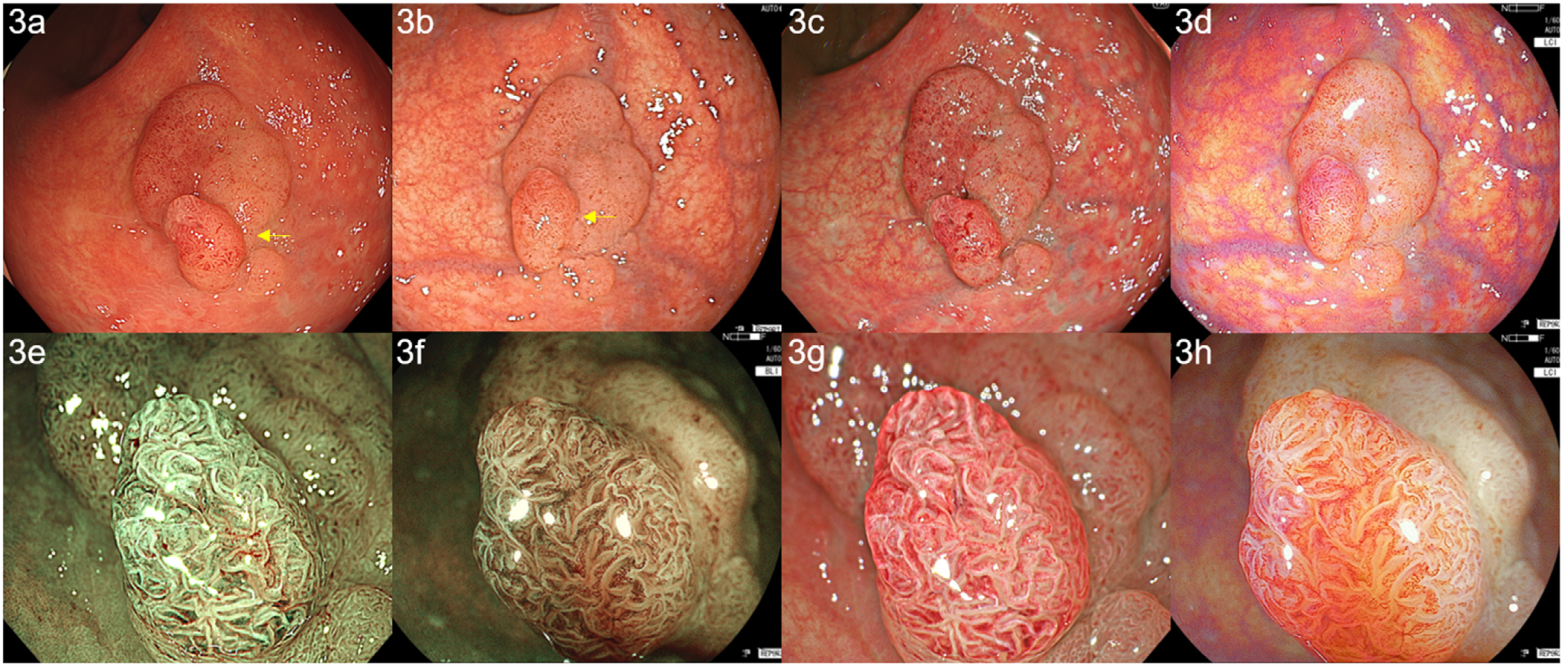
Images of adenoma obtained by color enhancement imaging (TXI) and linked color imaging (LCI) using the EVIS X1 (CF‐EZ1500DI) and ELUXEO systems, compared by tablet‐image comparison (TIC). (a) A superficial elevated lesion of 25 mm with a nodule on the surface was observed by white light imaging (WLI) using CF‐XZ1200I (low‐grade adenoma, rectum). (b) Based on TIC, WLI using the ELUXEO system (setting: H/+4/+4) produced an image that was less reddish and brighter in comparison to the EVIS X1 image. (c) In TXI, the redness of the nodule was emphasized. (d) In comparison to TXI, linked color imaging (LCI) was less reddish and brighter. (e) Magnified observation with narrow band imaging (NBI) clearly showed an irregular surface pattern. The vessel pattern was irregular and a little complex. (f) In blue light imaging (BLI), the surface pattern was less irregular and more enhanced in comparison to the EVIS X1 image. (g) In TXI, the surface pattern was irregular and complex, similar to NBI. (h) LCI magnified observation showed that the surface and vessel pattern were irregular, similar to BLI

The second case was a man in his 50s. Distant‐view observation with WLI using the CF‐XZ1200I (EVIS X1: structure enhancement setting: A5) revealed a superficial elevated lesion of 25 mm with a nodule on the surface (Figure 3a). In WLI using the ELUXEO system (setting: H/+4/+4) by TIC, the image was less reddish and brighter than the EVIS X1 image (Figure 3b). On TXI, the redness of the nodule was emphasized (Figure 3c). In comparison to TXI, LCI was less reddish and brighter (Figure 3d). Magnified observation of the nodule with NBI using EVIS X1 (structure enhancement setting: A8) showed an irregular surface pattern (Figure 3e). The vessel pattern was irregular and a little complex. An image of the same area with magnified BLI was more brownish and the surface pattern was less irregular and more enhanced in comparison to the EVIS X1 image (Figure 3f). The vessel pattern was slightly thicker than that on NBI. TXI magnified observation (TXI1) emphasized the redness (Figure 3g). The surface pattern was irregular and complex, similar to NBI. On LCI magnified observation, the surface and vessel pattern were irregular, similar to BLI (Figure 3h). We diagnosed this lesion as JNET 2B according to NBI and BLI and performed ESD. A histopathological examination revealed low‐grade adenoma. All the modalities in EVIS X1 and ELEXEO were used in Auto mode.

We calculated the color difference value and the brightness value of the tumors as an objective evaluation of TXI and LCI with software (Adobe Photoshop 2021; Adobe Inc., San Jose, USA) using the CIEL*a*b* color space and delta ELab formulas according to our previous report.^6^ The values of color difference value for TXI and LCI were 37.6 and 34.0 for the first case and 21.4 and 22.0 for the second case. The values of brightness value for TXI and LCI were 147.0 and 163.0 for the first case and 159.0 and 187.0 for the second case.

## DISCUSSION

Regarding the comparison of images obtained by two systems, we previously used an electronic medical report. Thus, we saw a report and remembered a situation and obtained images according to our memory. However, in most cases, we failed to obtain appropriate images for comparison. Then, we changed this method to use a printed image. However, this was cumbersome, especially in cases with large amounts of images. Finally, we developed our original method, TIC, which enabled the accurate comparison of images obtained by two modalities, as was demonstrated in this report.

Some papers already showed the efficacy of LCI for improving polyp detection.^7^, ^9^ On the other hand, no studies have reported the detectability of colorectal lesions on TXI. Thus, a detailed comparison between TXI and LCI could be performed by TIC. In these two cases, with a distant view, TXI showed greater redness than LCI. LCI showed slightly higher brightness than TXI. In magnified TXI and LCI, the irregularities observed were similar to NBI and BLI, respectively. Thus, we hypothesized that TXI and LCI magnification can be performed for tumor characterization in some cases. Regarding NBI and BLI, images obtained by NBI were a little different from those obtained by BLI and were more irregular and complex than images obtained by BLI. However, our previous paper showed a consistently high rate (81.7%) of JNET classification between NBI magnification (with the previous system) and BLI magnification, thus, NBI and BLI magnification produce a diagnosis that is almost the same.^10^ However, the previous system was used in the study. A large‐scale study using EVIS X1 should be performed to compare TXI with LCI and NBI with BLI.

In conclusion, we herein report our experience with two colonic neoplastic lesions in which we were able to precisely compare the endoscopic views of two LED colonoscopes, especially TXI and LCI, by TIC.

## CONFLICT OF INTEREST

Naohisa Yoshida and Osamu Dohi received a research grant from Fujifilm. All other authors declare no conflict of interest.

## FUNDING INFORMATION

The author Naohisa Yoshida and Osamu Dohi were supported by a research grant from Fujifilm (J162001222).

## ETHICAL STATEMENT

This case report was performed with the approval of the Ethics Committee of Kyoto Prefectural University of Medicine (ERB‐C‐1600) and was in accordance with the World Medical Association Declaration of Helsinki. We obtained a written informed consent from each case.
